# The complete mitochondrial genome of *Paracheirodon axelrodi* (Characiformes: Characidae) and phylogenetic studies of Characiformes

**DOI:** 10.1080/23802359.2019.1681307

**Published:** 2019-10-30

**Authors:** Yifan Liu, Fang Meng, Bingjian Liu, Youkun Huang, Qi Wang, Tao Zhang

**Affiliations:** a National Engineering Research Center for Marine Aquaculture, Zhejiang Ocean University, Zhoushan, China;; b National Engineering Laboratory of Marine Germplasm Resources Exploration and Utilization, Marine Science and Technology College, Zhejiang Ocean University, Zhoushan, China;; c Zhejiang Province Key Lab of Mariculture and Enhancement, Marine Fisheries Research Institute of Zhejiang, Zhoushan, China

**Keywords:** *Paracheirodon axelrodi*, mitochondrial genome, evolutionary relationships

## Abstract

The *Paracheirodon axelrodi* is an important freshwater fish in the Amazon basin of South America, more expensive than *Paracheirodon innesi*. Here, we describe the complete 17,100 base pair (bp) mitochondrial genome of *Paracheirodon axelrodi* mohavensis. The mitogenome has a nucleotide base composition of A (29.62%), T (29.41%), G (15.39%), and C (25.62%), and encodes 13 protein subunits, 22 tRNAs, a 12S rRNA of 952 bp and 16S rRNA of 1665 bp, and a 1433 bp D-loop control region, each located in the conserved mtDNA structure typical for Characidae fishes. All protein-coding genes have initiation codons of ATG, ND1, ND2, CO2, ND3, ND4L, ND5, ND6 and Cytb ended by TAA as a stop codon, ND4 ended by AGA as a stop codon, CO1 ended by AGG as a stop codon, ATP8 and CO3 ended by TAG as a stop codon, ATP6 ended by ATT as a stop codon. This characterised mitogenome may help inform management practices for *Paracheirodon axelrodi* mohavensis by facilitating future studies on how allopatric populations of this imperilled species are evolving across refuge habitats.

The *Paracheirodon axelrodi* is a species of the family Characiformes, which are distributed in slow-flowing rivers in Brazil, Colombia, and Venezuela (Mills and Vevers 1982; Beheregaray et al. 2010). *Paracheirodon axelrodi* has a certain difficulty in breeding, and the water temperature is stricter, more precious than other *Paracheirodon innesi*. Based on these characteristics, there were few reports about its basic biology data including genetic information. Identification of complete mitogenome of the *Paracheirodon axelrodi* would supplement the limited data on molecular level and be reference for systematics.

The specimen was collected from Amazon Basin, South America (15°31′5″S 71°45′55″W) and stored in a refrigerator of −80 °C in Zhejiang Engineering Research Centre for Mariculture and Fishery Enhancement Museum (Accession number: PA180217). Total genomic DNA was extracted from muscle tissue of individual using the phenol–chloroform method (Barnett and Larson 2012; Toni et al. 2018). The calculation of base composition and phylogenetic construction was conducted by MEGA6.0 software (Kumar et al. 1994; Tamura et al. 2013). Similar to the typical mitogenome of vertebrates, the mitogenome of *Paracheirodon axelrodi* is a closed double-stranded circular molecule of 17,100 bp (GenBank accession MH998225), which contains 13 protein-coding genes, 2 ribosomal RNA genes, 22 tRNA genes, and 1 main non-coding regions (Boore 1999; Zhu et al. 2018a; Zhu et al. 2018b). The overall base composition is 29.62%, 29.41%, 25.58%, and 15.39% for A, T, C, and G, respectively. Most mitochondrial genes are encoded on H-strand except for ND6 and eight tRNA genes (tRNA-Gln, tRNA-Ala, tRNA-Asn, tRNA-Cys, tRNA-Tyr, tRNA-Ser, tRNA-Glu and tRNA-Pro), which are encoded on the L-strand. All of them use the initiation codon ATG, which is quite common in vertebrate mtDNA (Miya et al. 2001; Liu et al. 2016). Most of them have TAA as the stop codon, whereas CO1 ends with AGG, ATP6 ends with ATT, ND4 ends with AGA, and two protein-coding genes (CO3 and ATP8) ended with TAG. The lengths of 12S rRNA located between tRNA^Phe^ and tRNA^Val^ and 16S rRNA located between tRNA^Val^ and tRNA^Leu^ were 952 bp and 1665 bp, respectively.

To explore the phylogenetic position of this *Paracheirodon axelrodi*, a phylogenetic tree was constructed based on the NJ analysis of 12 PCGs encoded by the heavy strand. The results of the present study supports *Paracheirodon axelrodi* has a closest relationship with *Prochilodus lineatus*, highly supported by a bootstrap value of 80 (Figure 1), which are consistent with the results based on morphology and other molecular methods.

**Figure 1.**
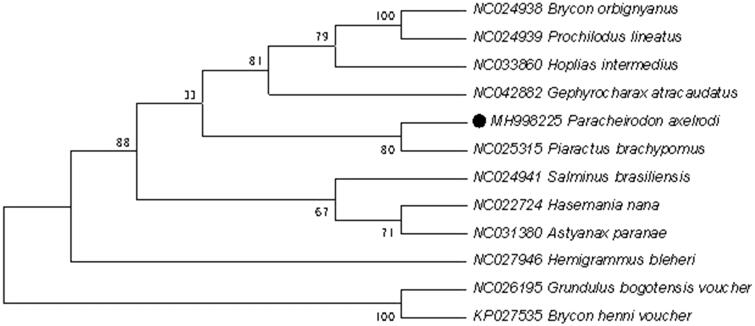
Neighbour-joining (NJ) tree of 12 Beloniformes species based on 12 PCGs encoded by the heavy strand. The bootstrap values are based on 1000 resamplings. The number at each node is the bootstrap probability. The number before the species name is the GenBank accession number. The genome sequence in this study is labelled with a black spot.
